# Spontaneous Bilateral Sternoclavicular Joint Septic Arthritis and Lumbar Discitis: An Unusual Case in a Healthy Adult

**DOI:** 10.1155/2017/7101694

**Published:** 2017-10-09

**Authors:** Georgios Mamarelis, Mohammad Zain Sohail, Athanasios Mamarelis, Hassan Fawi, Jehangir Mahaluxmivala

**Affiliations:** ^1^Princess Alexandra Hospital, Hamstel Road, Harlow CM20 1QX, UK; ^2^Queen's Medical Centre, Derby Rd, Nottingham NG7 2UH, UK

## Abstract

**Introduction:**

Septic arthritis of the sternoclavicular (SC) joint is a rare condition. Typically, it presents in patients with risk of infection and is usually unilateral. In this report, we describe a case of spontaneous bilateral sternoclavicular joint infection of an otherwise healthy adult.

**Case Presentation:**

A 67-year-old man presented in our hospital complaining of 2-week history of neck and chest pain which was radiating to his shoulders bilaterally. Clinical examination revealed erythema and swelling of the sternoclavicular area. Inflammatory markers were raised. Image investigation with CT and MRI was undertaken and verified the presence of bilateral sternoclavicular joint infection. The patient received prolonged course of intravenous antibiotics since his admission. The patient was discharged in a good condition and followed up in clinic.

**Conclusion:**

High index of clinical suspicion of SC joint infection is important for early diagnosis to avoid further complications.

## 1. Introduction 

Sternoclavicular joint septic arthritis (SCSA) represents only the 0.5 to 1% of all joint infections in the general population [1, 2]. The most common cause is* Staphylococcus aureus*, followed by* Pseudomonas* species [3–6]. We describe a rare case of bilateral SCSA in an otherwise healthy adult with no risk factor for infection.

## 2. Case Presentation

A 67-year-old man previously fit and well with a significant past medical history of hypertension and gout was admitted with a two-week history of fatigue and chest and lower back pain. He was septic and tender over the sternoclavicular area, raising suspicion of SCSA. Detailed exploration of the history did not identify any risk factors for SCSA such as diabetes mellitus, intravenous drug abuse, trauma, or vascular heart disease.

**Figure 1:**
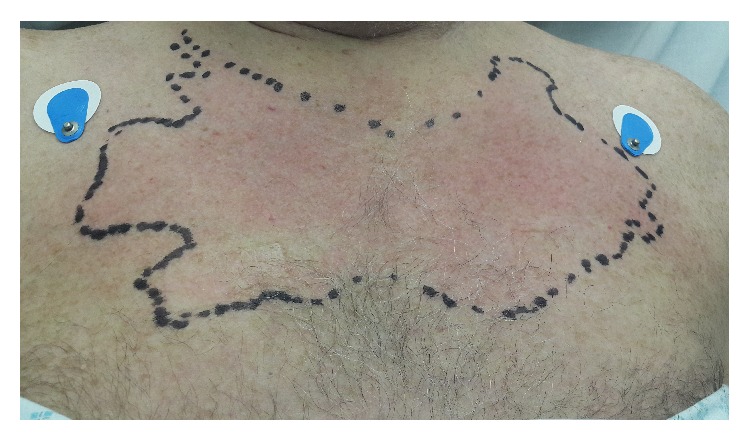
Erythema around bilateral sternoclavicular area in a “butterfly-like” distribution.

Physical examination revealed a temperature of 38.3 Celsius with the rest of the observations being within normal limits. Bilateral sternoclavicular joints were moderately swollen and tender with an erythematous area in a “butterfly-like” distribution (Figure 1). He had diffuse lower back pain, without neurological deficit. The rest of the systemic examinations, including the lymphoreticular and cardiovascular examinations, were unremarkable.

Haematological investigations at admission revealed raised inflammatory markers. Two blood cultures taken 48 hours apart yielded heavy growth of* Staphylococcus aureus*, sensitive to flucloxacillin and rifampicin. Autoimmune profile and virology screen were normal. Echocardiography was essentially unremarkable and did not yield any vegetation.

**Figure 2:**
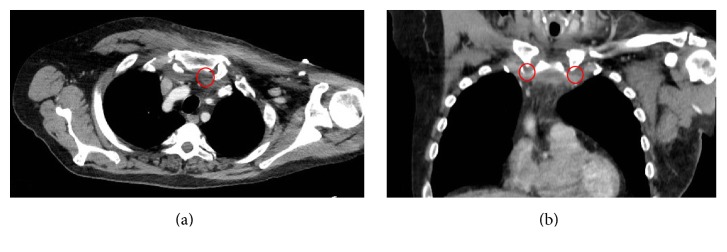
Chest CT scans. (a) Axial image; (b) coronal image showing evidence of bilateral sternocleidomastoid inflammation/infection. Thickened soft tissue was noted surrounding the SC joints and posterior to the sternum labelled by the red circle.

Plain radiographs of the chest and clavicle were reported as normal. Computed tomography (CT) scan of his chest showed evidence of bilateral sternocleidomastoid inflammation/infection. Thickened soft tissue was noted surrounding the SC joints and posterior to the sternum (Figure 2).

**Figure 3:**
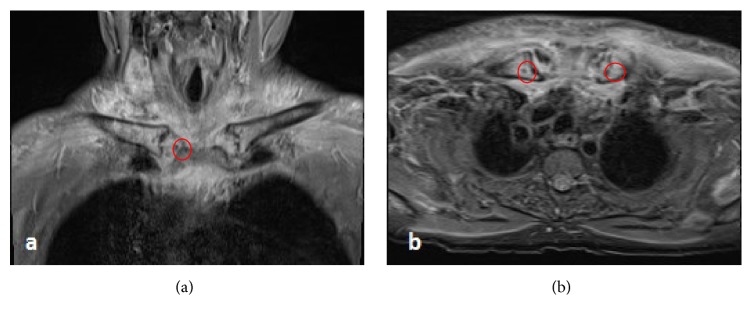
Sternoclavicular joints MRI scan STIR sequence. (a) Coronal image; (b) axial image showing moderate bone marrow edema in the medial third of the clavicles bilaterally, extending to the subarticular region. There was fluid signal in the SCJ space with mild to moderate marrow edema seen in the manubrium. No radiological signs of osteomyelitis were noted.

**Figure 4:**
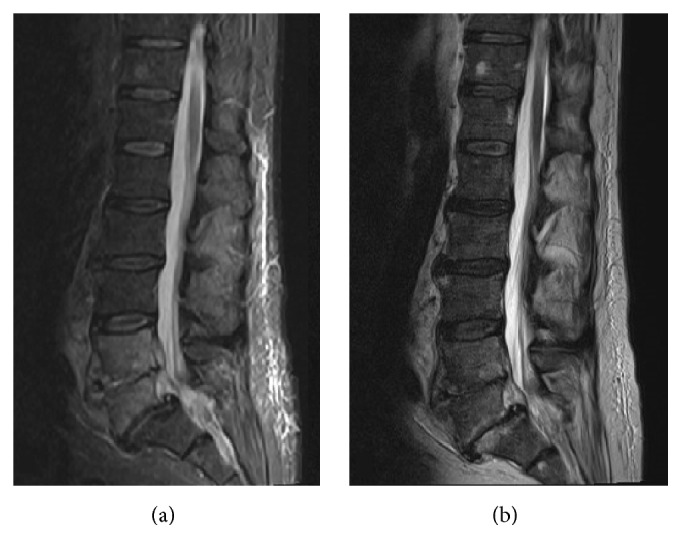
Lumbar spine MRI scan. (a) STIR sequence; (b) T2 sequence.

Magnetic resonance imaging (MRI) scan of the sternoclavicular joints (SCJ) has shown moderate bone marrow edema in the medial third of the clavicles bilaterally, extending to the subarticular region. There was fluid signal in the SCJ space with mild to moderate marrow edema seen in the manubrium. No radiological signs of osteomyelitis were noted (Figure 3). MRI scan of his lower back revealed features consistent with L4/5 discitis (Figure 4).

The patient was started on intravenous (IV) flucloxacillin and oral rifampicin. Patient responded well to the treatment and the inflammatory markers improved on the 4th day of admission.

SC joint aspiration was inapplicable as the amount of the fluid was not significant on the MRI scan.

The patient was hospitalized for two weeks because he was unable to mobilize due to chest pain. He was discharged in good condition with an additional 4-week course of IV flucloxacillin. Two weeks later on his scheduled follow-up, there was persistent bilateral SC joint pain. Anti-inflammatory medication was recommended. The pain gradually improved and after eight weeks he was able to mobilize pain-free with an uneventful recovery.

## 3. Discussion

Sternoclavicular joint septic arthritis is very rare. It involves only the 0.5–1% of all joint infections [2, 7]. Immunocompromised and chronically ill patients, such as diabetics, intravenous drug abusers, those on long-term steroids, and chronic renal failure, are most susceptible [1].

In this case, we report an otherwise healthy adult who presented with bilateral SCSA and L4/5 discitis. There were no predisposing risk factors or any evident source of infection. According to Ross and Shamsuddin, predisposing risk factors are intravenous drug user (21%), distant site of infection (15%), diabetes mellitus (13%), trauma (12%), and infected central venous line (9%). Up to 23% of the SCSA patients had no risk factors [2]. Bar-Natan et al. reported that SCSA occurs in less than 0.5% of the healthy population and the route of infection is unknown in most of the cases [1].

SCSA usually presents with fever and pain on the neck and anterior chest, which can radiate to the shoulders. Erythema and swelling of the skin over the sternoclavicular area is common [2, 8]. We report an interesting aspect of bilateral SCSA with a characteristic “butterfly-like” erythema.

Fowler Jr. et al. demonstrated four clinical characteristics to predict a complicated infection. The most important of these were positive follow-up culture results at 48–96 hours. The remaining three characteristics were community acquisition, skin examination findings suggesting acute systemic infection, and persistent fever at 72 hours [9]. Our patient had low chance of complicated infection as fever was resolved in less than 72 hours and his blood cultures were negative in 96 hours.

In SCSA, blood cultures are positive in 13% of patients. Surgical debridement and needle aspiration are positive in 36% and 77% accordingly [2].

CT or MRI scan is the image investigation of choice to define the severity of the infection and guide the surgical plan if needed [2, 10]. Bodker et al. proposed that MRI should be the initial image investigation of SCSA [11]. In 55% of all cases, osteomyelitis of the distal third of clavicle or the manubrium or both may be present [12].

The majority of early SCJ septic arthritis will resolve with conservative treatment, considering that there is no abscess formation or mediastinum spread [2, 13]. The average time of intravenous antibiotics should be 52 to 70 hours [2].

Surgical intervention with debridement and intravenous antibiotics should be considered in cases with abscess development. Occasionally, excision of the medial end of the clavicle, first rib, and manubrium is obligatory. In these cases, the chest wall defect is being covered by a rotational flap of the pectoralis major muscle or an advancement flap [14–16].

The combination of unilateral SCSA and discitis has been described in the literature [17–19]. The most common way for spontaneous pyogenic spondylodiscitis to spread is usually hematogenous from infections of the skin, subcutaneous tissues, or urinary tract [20].* Staphylococcus aureus* is the most common causative microorganism, followed in by* Brucella*,* Salmonella*, and* Mycobacterium tuberculosis* [21, 22]. It is likely that the patient in our case had discitis initially and then hematogenously spread to the bilateral sternoclavicular joints.

## 4. Conclusion

SCJ infections are very rare. High degree of clinical suspicion is required as the equivocal symptoms of neck, chest, and shoulder pain may mask the initial diagnosis. Physicians should suspect SCSA in patients with chest pain and high fever, even in the absence of risk factors, as demonstrated in our case. Systemic examination is essential to rule out other possible sources of infection, like discitis.

In our case, we emphasize the appearance of a characteristic “butterfly-like” erythema as a sign of high index of clinical suspicion in bilateral SCSA. Surgical intervention is often required; however, in our patient, the SCSA resolved with intravenous and oral antibiotics and no major intervention was necessitated.
